# Acroparesthesia in a Female: Diagnostic Dilemma

**DOI:** 10.1155/2014/172197

**Published:** 2014-11-02

**Authors:** Fnu Kelash, Lara Kujtan, Padmaja V. Mallidi

**Affiliations:** Carilion Clinic Virginia, 1906 Belleview Ave SE, Roanoke, VA 24014, USA

## Abstract

Fabry disease is an X-linked lysosomal storage disorder caused by deficient activity of a-galactosidase A (also known as ceramide trihexosidase) and resultant accumulation of globotriaosylceramide (Gb3) and related glycophospholipids. The disease affects nearly all major organ systems, with the primary sites damaged by Gb3 including renal glomeruli, myocardium, neurons of the dorsal ganglion and autonomic nervous system, and vascular endothelial and smooth muscle. Progressive deposition in these organ systems leads to renal and heart failure; debilitating pain as a result of nervous system involvement also occurs.

## 1. Case Report

A 23-year-old Caucasian female presented with unusual symptoms since the age of 18. At that time, she began to note pain, tightness, and a burning sensation of her fingers and toes, with worse involvement of the former. These symptoms worsened with exertion, with warm temperatures, while holding a glass of wine, and when being measured with a sphygmomanometer at her primary care physician's office. The symptoms usually lasted for 30 to 40 minutes, with slow recovery and no residual symptoms. Elevation of the arms and legs as well as cooler temperatures occasionally resulted in symptom relief. She also noticed decreased sweat production.

She denied bowel or bladder incontinence, pruritus, muscle weakness, imbalance, visual changes, or slurred speech.

Her medical history also included acne treated with vibramycin, as well as vitamin D deficiency treated with vitamin D supplementation. She took no other medications and had no known drug allergies. Her family history was significant for a maternal grandmother with diabetes mellitus, cataracts, and hearing loss, as well as a maternal uncle with renal disease. Her only hospitalization occurred at the age of 19 after presenting with syncope, for which she underwent extensive evaluation. CT scan of her brain was negative for acute abnormalities. Cardiology was consulted; however EKG was normal and telemetry monitoring did not reveal any underlying arrhythmias.

After initial evaluation by her primary care physician demonstrated an unremarkable physical examination, normal chemistries, and complete blood counts, she was referred to a vascular surgeon. An arterial duplex study was negative for any abnormalities. Her painful episodes persisted and increased in frequency, resulting in debilitating pain affecting her quality of life and impairing her ability to work as a school teacher. She was then referred to both a neurologist and a rheumatologist. Further laboratory testing included antinuclear antibody titers, erythrocyte sedimentation rate, uric acid, phosphorus, and liver function tests and was all within normal limits.

She was advised that stress was the likely etiology of her underlying symptoms.

She was finally referred to a hematologist for evaluation of possible erythromelalgia, although she did not experience pruritus. Her symptoms were found to be consistent with acroparesthesias and hypohydrosis. This combined with her otherwise negative work-up, as well as her family history of hearing loss and renal disease, raised suspicion for Fabry disease.

She was found to have a galactose-alpha-1,3-galactose level <0.10 suggestive of possible Fabry disease. This was followed by genetic analysis, which demonstrated that she was a heterozygous carrier of Fabry disease with a deletion mutation in Exon 3, C427G>A (Ala14Thr). She was started on enzyme replacement therapy, with improvement in her symptoms.

## 2. Discussion

Fabry disease is a rare X-linked lysosomal storage disease; the incidence of the classic phenotype is 1 : 40,000–60,000 in males. It is also known as Anderson-Fabry disease, angiokeratoma corporis diffusum, angiokeratoma, and hereditary dystopic lipidosis.

The mutation results in deficient activity of a-galactosidase A (also known as ceramide trihexosidases) and resultant accumulation of globotriaosylceramide (Gb3) and related glycophospholipids [1]. The variable clinical presentations in heterozygous female carriers are related to the a-galactosidase A activity levels.

The accumulation of Gb3 affects nearly all major organ systems, including renal glomeruli and tubular epithelial cells; myocardial cells and valvular fibrocytes; neurons of the dorsal ganglion and autonomic nervous system; and endothelial, perithelial, and smooth muscle cells of the vascular system. Progressive deposition of Gb3 in these organ systems leads to renal and heart failure, as well as debilitating pain as a result of nervous system involvement [2].

Symptoms classically begin in childhood or adolescence with the appearance of a reddish to dark blue skin rash known as angiokeratoma [3], decreased or absent sweat production (hypohydrosis or anhydrosis), and discomfort with warm temperatures.

Pain is an early symptom, usually presenting as episodic severe pain in the hands and feet known as acroparesthesias. This occasionally occurs in the arms and legs [4]. These episodes may last for hours to days and are frequently associated with exercise, fatigue, and/or fever.

Major morbidity in Fabry disease is due to renal involvement, which can vary from asymptomatic proteinuria to progressive renal failure; cardiovascular disease including syncope, cardiomyopathy, valvular heart disease, and ischemic heart disease [1]; central nervous system involvement such as dizziness, memory impairment, depression, and stroke [2]; and psychiatric manifestations such as depression and insomnia.

Serum a-galactosidase A level can be measured on a dried blood spot, and in males low level is sufficient for diagnosis. In females, genetic testing is required because serum a-galactosidases A level may be normal [5].

Enzyme replacement therapy is safe and can reverse substrate storage in the lysosome. It has proven benefit in decreasing disease progression and alleviating the symptoms associated with Fabry disease. Supportive treatments and medications are used for concomitant systemic disease manifestations [6].

## 3. Conclusion

Fabry is rare X-linked lysosomal storage disease with variable symptoms in heterozygous carrier females and affected males, including acroparesthesias; skin manifestations such as cherry angiomas, angiokeratomatosis, anhydrosis, hypohydrosis, and telangiectasias; renal failure, central nervous system manifestations, and cardiovascular disease.

It should be considered early in the presentation of acroparesthesias and skin manifestations, as it can easily be treated with enzyme replacement therapy, which prevents major complications such as the onset of renal and cardiac disease, as well as preventing debilitating pain syndromes.
